# Post-Transplant Thrombotic Microangiopathy due to a Pathogenic Mutation in Complement Factor I in a Patient With Membranous Nephropathy: Case Report and Review of Literature

**DOI:** 10.3389/fimmu.2022.909503

**Published:** 2022-05-26

**Authors:** Maryam Saleem, Sana Shaikh, Zheng Hu, Nicola Pozzi, Anuja Java

**Affiliations:** ^1^ Division of Nephrology, Department of Medicine, Washington University School of Medicine, St. Louis, MO, United States; ^2^ Division of Nephrology, Department of Medicine, University of California, San Francisco, San Francisco, CA, United States; ^3^ Division of Rheumatology, Department of Medicine, Washington University School of Medicine, St. Louis, MO, United States; ^4^ Department of Biochemistry and Molecular Biology, Edward A. Doisy Research Center, Saint Louis University School of Medicine, St. Louis, MO, United States

**Keywords:** thrombotic microangiopathy, kidney transplantation, complement factor I, membranous nephropathy, atypical hemolytic uremic syndrome, complement functional analysis

## Abstract

Thrombotic microangiopathy (TMA) is characterized by microangiopathic hemolytic anemia, thrombocytopenia and organ injury occurring due to endothelial cell damage and microthrombi formation in small vessels. TMA is primary when a genetic or acquired defect is identified, as in atypical hemolytic uremic syndrome (aHUS) or secondary when occurring in the context of another disease process such as infection, autoimmune disease, malignancy or drugs. Differentiating between a primary complement-mediated process and one triggered by secondary factors is critical to initiate timely treatment but can be challenging for clinicians, especially after a kidney transplant due to presence of multiple confounding factors. Similarly, primary membranous nephropathy is an immune-mediated glomerular disease associated with circulating autoantibodies (directed against the M-type phospholipase A2 receptor (PLA2R) in 70% cases) while secondary membranous nephropathy is associated with infections, drugs, cancer, or other autoimmune diseases. Complement activation has also been proposed as a possible mechanism in the etiopathogenesis of primary membranous nephropathy; however, despite complement being a potentially common link, aHUS and primary membranous nephropathy have not been reported together. Herein we describe a case of aHUS due to a pathogenic mutation in complement factor I that developed after a kidney transplant in a patient with an underlying diagnosis of PLA2R antibody associated-membranous nephropathy. We highlight how a systematic and comprehensive analysis helped to define the etiology of aHUS, establish mechanism of disease, and facilitated timely treatment with eculizumab that led to recovery of his kidney function. Nonetheless, ongoing anti-complement therapy did not prevent recurrence of membranous nephropathy in the allograft. To our knowledge, this is the first report of a patient with primary membranous nephropathy and aHUS after a kidney transplant.

## Introduction

Atypical hemolytic uremic syndrome (aHUS) is a classic complement-mediated thrombotic microangiopathy (TMA) resulting from inadequately controlled activation of the alternative pathway (AP) of the complement system (1, 2). The etiology of aHUS is commonly a heterozygous, loss-of-function mutation in a regulator (Factor H, Factor I or Membrane Cofactor Protein). Less commonly a gain-of-function mutation in a complement activator (C3 or Factor B) may be identified (3). A TMA after a kidney transplantation can be *de novo* or recurrent (4, 5). Patients with recurrent TMA almost always have a complement-mediated disease. However, *de novo* TMA may be complement-mediated or secondary to transplantation-associated triggers such as immunosuppressive medications, ischemia reperfusion injury, viral infections, malignancy or antibody-mediated rejection. *De novo* TMA is reported in 1-15% patients, although the true frequency is unknown, and the implication of a dysregulated complement system may be underestimated.

Primary membranous nephropathy (MN) is an autoimmune-mediated glomerular disease and is one of the most common causes of nephrotic syndrome in adults (6). The disease may recur after kidney transplantation in 35-40% cases or occur as a *de novo* form. Over the last few years, several different podocyte antigens have been identified in association with MN, such as M-type phospholipase A2 receptor (PLA2R), thrombospondin type-1 domain-containing 7A, exostosin 1 and exostosin 2, NELL-1 and, most recently, protocadherin FAT1 (7, 8). Evidence from human and animal data has suggested that the complement system may play a role in the pathogenesis of MN, however, there has been substantial heterogeneity in the complement activation profiles reported in patients. One reason for the variation in the extent of complement activation may be due to differences in the subclass of IgG antibodies associated with the various antigens. We report a unique case of complement mediated *de novo* TMA (aHUS) in a patient after kidney transplantation who had an underlying diagnosis of PLA2R+ MN as the etiology of his native kidney disease. The patient responded successfully to anti-complement therapy without relapse of the TMA but developed early and aggressive recurrence of the membranous nephropathy in the allograft.

## Case Description

### Patient Information

A 28-year-old African American male with end stage renal disease (ESRD) secondary to biopsy-proven membranous nephropathy (PLA2R antibody positive) underwent a 2A, 2B, 1DR mismatch, ABO incompatible living-unrelated kidney transplant. There was no history of kidney disease in the family. Due to the ABO incompatibility (donor A+, recipient O+ with an anti-A antibody titer of 64), he was treated with 10 sessions of plasmapheresis, rituximab and mycophenolic acid prior to transplant (per our center’s protocol) with a decrease in the anti-A antibody titer to 4. Induction immunosuppression included methylprednisolone (7 mg/kg) and thymoglobulin (6 mg/kg).

### Clinical Findings and Diagnostic Assessment

**Figure 1:**
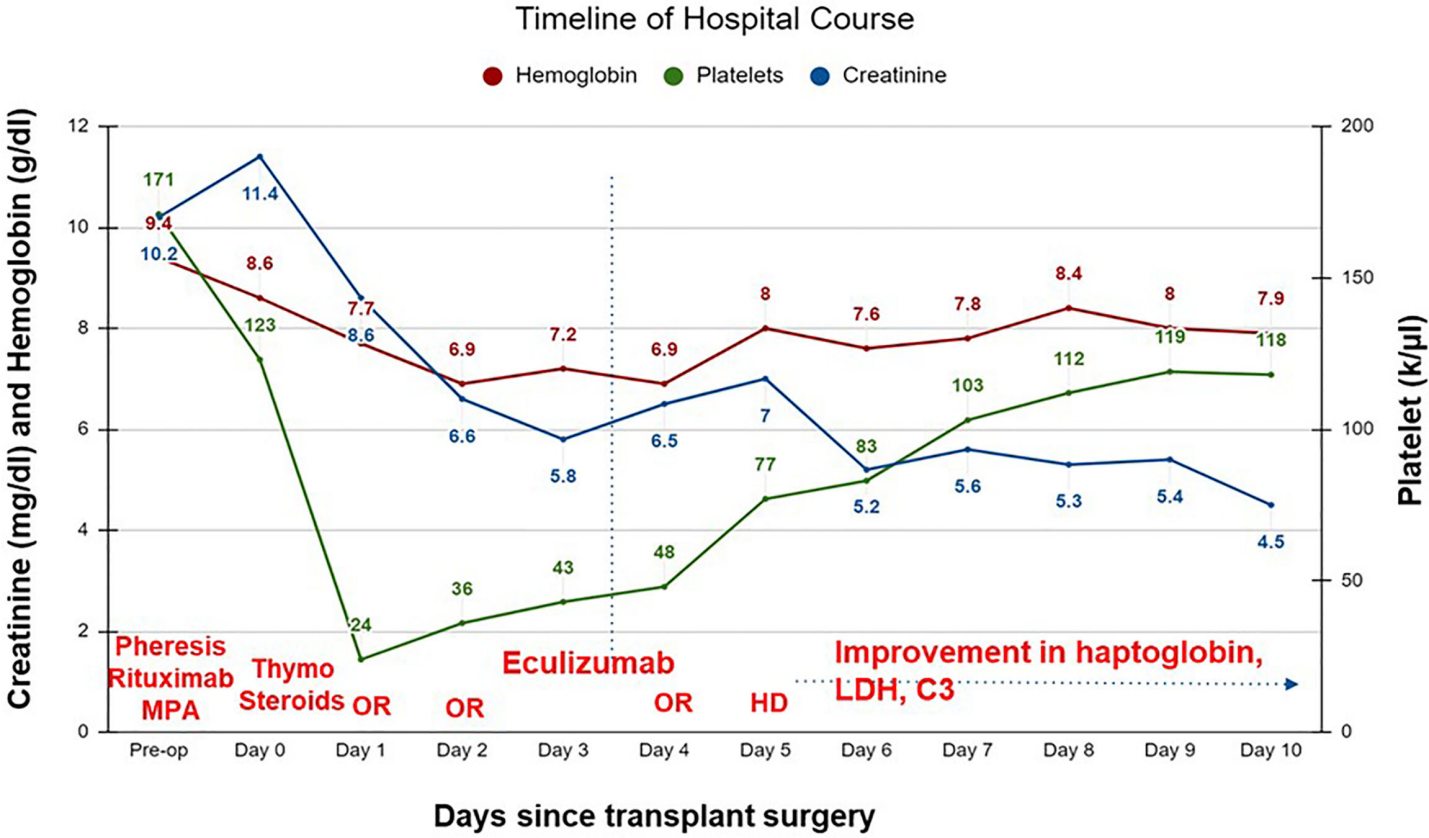
Timeline of hospital course of patient after a living unrelated kidney transplantation. C3, complement 3; HD, hemodialysis; LDH, lactate dehydrogenase; OR, operative room; thymo, thymoglobulin; MPA, mycophenolic acid.

On postoperative day (POD) 1, the patient developed increased bleeding from the surgical incision site and was taken back to the operating room (OR) for exploration and washout. Diffuse oozing was noted with a hematoma in the retroperitoneum. Laboratory data were notable for anemia (hemoglobin 6.9-7.7 g/dL; reference range 13-17 g/dL), severe thrombocytopenia (platelet count 24-36 k/µL; reference range 150-400 k/µL), low haptoglobin (<10 mg/dL; reference range 30-200 mg/dL) and high lactate dehydrogenase (580-736 units/L; reference range 150-250 units/L) **(** **Figure 1** **).** Additional work-up revealed low C3 (58 mg/dL; reference range 90-180 mg/dL) and a low normal C4 (12.9 mg/dL; reference range, 10-40 mg/dL). ADAMTS13 (a disintegrin and metalloproteinase with a thrombospondin type 1 motif, member 13) activity and coagulation profile were normal. This raised concern for a TMA. Genetic testing for complement variants was sent.

### Therapeutic Intervention

**Figure 2:**
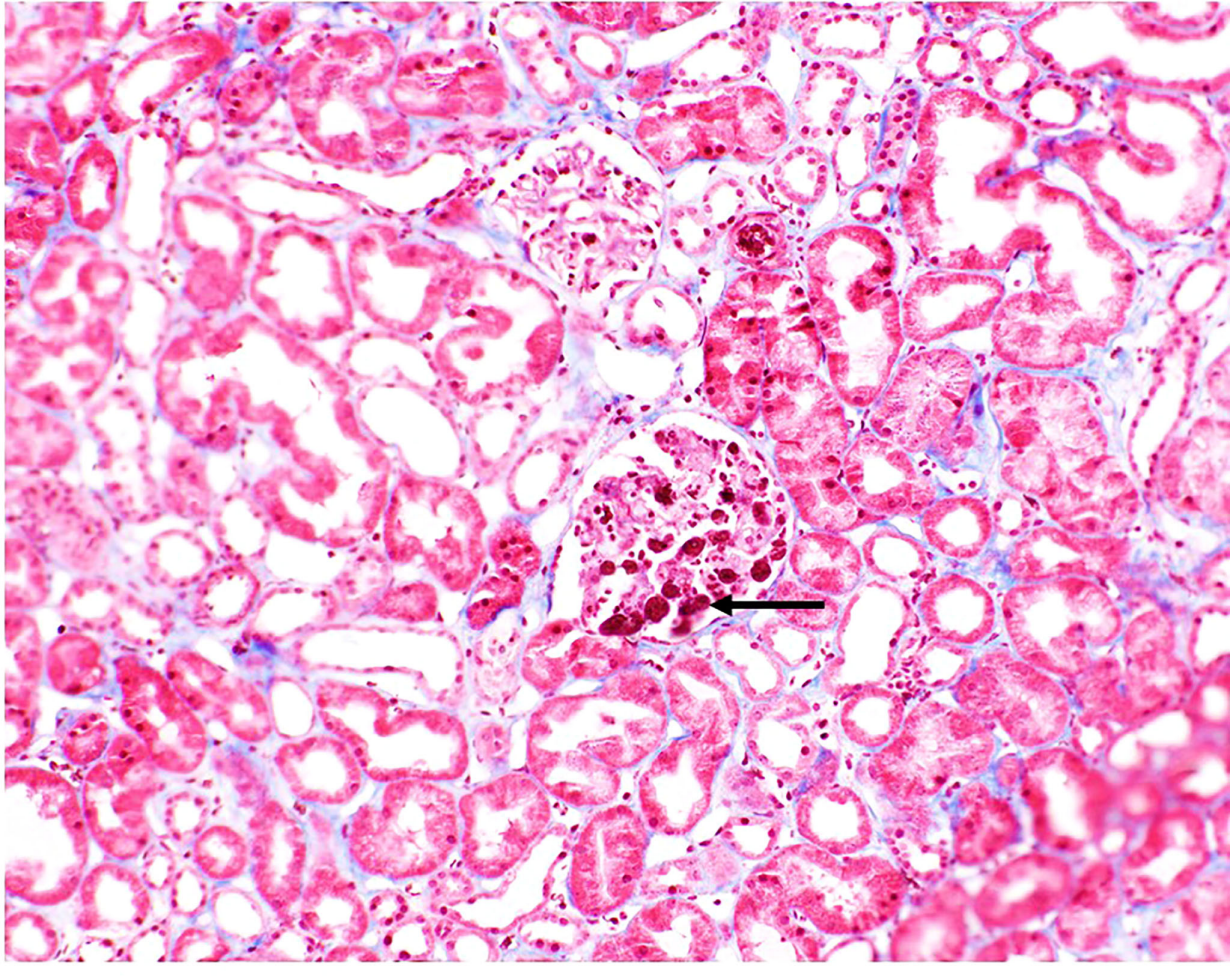
Biopsy image from patient. Fibrin thrombi seen in glomeruli (black arrow). Small arteries and arterioles demonstrated focal fibrinoid necrosis of the arterial wall (not shown).

Tacrolimus was not initiated. Eculizumab was administered on POD 3. Patient returned to the OR on POD 4 for revision and closure and at that time an intraoperative biopsy was performed which confirmed a TMA with no evidence of acute cellular or antibody-mediated rejection (**Figure 2**).

### Follow-Up and Outcomes

The patient remained oliguric and required hemodialysis on POD 5. Over the next 24 hours, signs of clinical recovery were evident with normalization of haptoglobin (86 mg/dL), improvement in lactate dehydrogenase (364 units/L) and C3 (112 mg/dL). Renal function improved and he did not require further dialysis. Serum creatinine on the day of discharge (POD 10) was 4.5 mg/dL. Eculizumab was continued as an outpatient. Creatinine stabilized at 1.8 mg/dL by POD 14, with no recurrence of TMA; however, the patient developed recurrent biopsy-proven membranous nephropathy a month later.

### Genetic Variant Analysis

**Figure 3:**
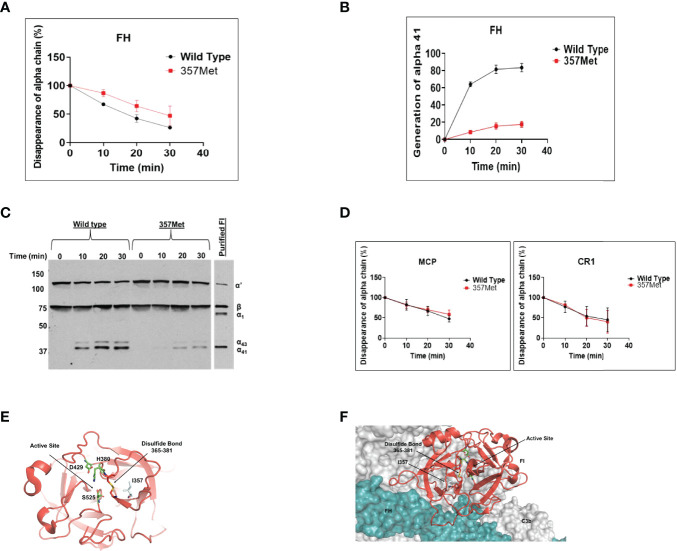
Functional evaluation of Factor I (FI) variant Ile357Met: proteolytic activity. The fluid-phase C3b proteolytic activity of the variant factor I (357Met) with its cofactor proteins (Factor H [FH], membrane cofactor protein [MCP], or complement receptor 1 [CR1]) was assessed by cleavage of purified C3b to iC3b and compared to wild type (WT). For these assays, purified WT and FI variant proteins were diluted in physiologic salt (150 mM NaCl) buffer with C3b (10 ng; Complement Technologies, Inc, Tyler, TX USA) at 37°C. Concentrations of WT or variant FI used with the individual cofactors were 10 ng with MCP, 20 ng with FH and 15 ng with CR1. Concentration of cofactor used in the reactions were 100 ng MCP, 200 ng FH or 150 ng CR1. Reactions were carried out in a total volume of 15 µl/reaction at 37°C. Kinetic analysis of the WT and variants was achieved through collection of sample at 0, 10, 20 and 30 min. At each time point 7 µl of 3x Laemmli reducing sample buffer was added to individual reactions to stop the reaction and then heated at 95°C for 5 min. The samples were electrophoresed on 10% Tris-glycine gel and then transferred to nitrocellulose for WB analysis. Membranes were rinsed with TBS-T (0.05%Tween-20) for 5 min and blocked overnight with 5% nonfat dry milk in PBS. Blots were probed with a 1:5,000 dilution of goat anti-human C3 (Complement Technologies, Inc, Tyler, TX, USA) followed by HRP-conjugated rabbit anti-goat IgG and developed with SuperSignal substrate (Thermo Fisher Scientific, Waltham, MA, USA). The signal detected on radiographic films was scanned using a laser densitometer (Pharmacia LKB Biotechnology, Piscataway, NJ, USA). Multiple exposures were used to establish linearity. **(A, B)**. The percentage of α’ chain remaining and generation of α41 fragment indicates cleavage of C3b to iC3b. Cleavage rate was measured by densitometric analysis of the α’ chain remaining as well as generation of α41 relative to the β chain. Data represent 2 separate experiments with bars corresponding to the standard error of mean (SEM). Upon comparison to WT FI, the proteolytic activity of variant 357Met was defective with FH. The P value for the difference in the percentage of α’chain remaining between WT and variant was 0.05 and for the difference in the percentage of α41 generation was <0.05. **(C).** Representative WB demonstrating cofactor activity of Factor H with the variant (357Met) compared with wild type FI as well as purified FI **(D)** No defect was observed with MCP or CR1 as the cofactor protein. I, isoleucine; M, methionine. Structural evaluation of Ile357Met **(E)** Mapping of Ile357 on the structure of FI shows that it is located in the serine protease domain of FI which harbors the catalytic activity. Although the variant is away from the catalytic serine (S525)), the substitution of I to a bulkier amino acid M likely alters the position of the disulfide bond 365-381, thus affecting the FI activity. **(F)** Mapping of the Ile357 on the triple complex of Factor H and C3b shows that the variant is away from the binding surface of FH, therefore we speculate that it likely leads to a conformational change resulting in low functional activity.

Genetic testing was conducted by the Genomic and Pathology services at Washington University in St. Louis and revealed a ‘variant of uncertain significance’ in Complement Factor I (*CFI)* (Ile357Met). This variant is located in the serine protease domain of FI which contains the catalytic site and has been reported at a frequency of 0.004% in population databases (gnomAd). We produced the variant protein recombinantly and conducted functional and structural analysis to define the significance of this variant using the methods described previously (9). As assessed by ELISA, the secretion of the recombinant protein by 293T cells compared to wild type (WT) was reduced [WT, 11.44 µg/ml ± 1.4 (standard error of mean); 357Met, 4.79 µg/ml ± 0.401(standard error of mean)]. However, patient’s serum antigenic level of Factor I (FI) was normal (3.6 mg/dL, reference range for Blood Center of Wisconsin laboratory is 2.4-4.9 mg/dL). Although these results raised the question whether decreased secretion of FI *in vitro* in 293T cells accurately translates to low antigenic levels in the patient, the variant Ile357Met has been reported previously in patients with low levels (10). Therefore, we speculated that the normal serum level in our patient was likely reflective of an increase in the secretion of the WT allele (indicative of the acute phase nature of FI). Additionally, functional analysis demonstrated that the variant had defective complement regulatory activity with Factor H (**Figures 3A– C**) but no defect was seen with membrane cofactor protein or complement receptor 1 (**Figure 3D**).

Structural analysis showed that Ile357 was located 8 Å away from the catalytic serine (S525) and 3.5 Å away from the conserved disulfide bond (365–381). The disulfide bond plays a vital role of keeping the catalytic histidine (H380) in place for optimal enzymatic activity (**Figure 3E**). Given that Met is bulkier and less hydrophobic than Ile, the substitution of Ile to Met likely alters the position of the disulfide bond 365-381, thus lowering the FI activity. We also mapped the variant on the triple complex with FH and C3b (**Figure 3F**) (11) and it does not seem to lay close to FH, therefore, we speculate that the variant likely causes a conformational change that results in reduced functional activity. These analyses established that the *CFI* Ile357Met variant was deleterious (due to both decreased secretion and functional activity) and thereby consistent with the diagnosis of aHUS in our patient.

## Discussion

Thrombotic microangiopathy (TMA) characterized by over-activation and dysregulation of the alternative pathway (AP) of complement cascade is called a primary TMA or aHUS. The clinical outcome of aHUS is unfavorable, typified by progression to ESRD and relapse after kidney transplantation, if not diagnosed and treated timely. aHUS may be mimicked by other disease processes, currently classified under secondary TMA, including infections, pregnancy, autoimmune conditions, and graft rejection. Kidney transplantation poses a problematic setting since there are multiple potential triggers (transplant surgery, drugs, rejection, and infections) for TMA development (12, 13). Consequently, it can be ‘tricky” for transplant clinicians to distinguish aHUS from these secondary TMAs.

Our case is a prime example of such a challenging scenario. The patient had a history of ESRD secondary to biopsy-proven primary MN and manifested a *de novo* TMA after kidney transplantation. After a systematic work-up, the etiology of the TMA was determined to be a pathogenic mutation in *CFI*. We speculate that the genetic mutation in *CFI* conferred a low risk of TMA in the native kidneys, and that he developed an early and aggressive disease after kidney transplantation due to the multiple additional risk factors (transplant surgery, ischemia-reperfusion injury, etc) that predisposed him to endothelial injury. Our strategy of recombinant protein production followed by detailed functional assessment defined the functional repertoire of the *CFI* variant protein (demonstrating that it was defective due to both low secretion and low function of the protein) and ascertained the diagnosis of complement-mediated TMA or aHUS in this patient and helped to differentiate it from a secondary TMA after kidney transplantation. Further it provided critical guidance relative to the underlying pathophysiology and appropriate therapeutic regimen.

We also considered the possibility that complement dysregulation due to the *CFI* genetic variant could have played a role in the etiology of membranous nephropathy. This speculation stems from the Heymann nephritis rat model that showed that subepithelial immune deposits initiate complement activation leading to C5b-9-mediated damage of the podocytes (14, 15). This has been further validated in human MN with evidence of C3 breakdown product deposition (C3c and C3d) on immuno-histologic staining and presence of C5b-9 in the urine (16). Although, the exact role of complement in MN and the predominant pathway involved remains unclear, several levels of evidence implicate the AP or lectin pathway. The absence of classical pathway components (C1q and C4) in glomeruli, IgG4 being the major subclass associated with PLA2R and the decrease in complement receptor 1 (CR1) expression on the podocyte observed in patients with primary MN have all led to the speculation that the AP activation may be dominant (17–20). There is also one case in the literature of MN in association with Factor H-autoantibodies in the absence of a TMA (21). These data indicate that a subset of patients with primary MN may have dysregulation of the AP and benefit from anti-complement therapy. Our patient developed recurrent MN within a month after transplantation despite being on eculizumab but did not develop recurrent TMA. Therefore, we believe that the MN in our patient was likely not complement-mediated, and he had two different primary immune processes (MN and aHUS) in the allograft. These two diseases have not been reported together before either in the native kidney or after a transplantation. Despite the discovery of multiple new antigens in MN, their precise role in complement activation remains unclear and more research is needed to better define the underlying pathogenic mechanisms of these antigens and to determine if and who would benefit from anti-complement therapy.

## Data Availability Statement

The original contributions presented in the study are included in the article/supplementary material. Further inquiries can be directed to the corresponding author.

## Ethics Statement

The studies involving human participants were reviewed and approved by Institutional review board, Washington University School of Medicine, St. Louis, MO. The patients/participants provided their written informed consent to participate in this study. Written informed consent was obtained from the individual(s) for the publication of any potentially identifiable images or data included in this article.

## Author Contributions

MS, SS, and AJ drafted the manuscript. ZH performed the experiments. NP conducted the structural analysis. SS and AJ prepared the figures. AJ edited the manuscript. All authors contributed to the article and approved the submitted version.

## Funding

Supported in part by Barnes Jewish Hospital Foundation Fund, Division of Nephrology, Washington University School of Medicine in St. Louis (AJ).

## Conflict of Interest

The authors declare that the research was conducted in the absence of any commercial or financial relationships that could be construed as a potential conflict of interest.

## Publisher’s Note

All claims expressed in this article are solely those of the authors and do not necessarily represent those of their affiliated organizations, or those of the publisher, the editors and the reviewers. Any product that may be evaluated in this article, or claim that may be made by its manufacturer, is not guaranteed or endorsed by the publisher.
